# Two-Year Weight Loss Outcomes After Sleeve Gastrectomy and Roux-en-Y Gastric Bypass in a Standardized Multidisciplinary Program: A Comparative Study

**DOI:** 10.7759/cureus.110737

**Published:** 2026-06-12

**Authors:** Jesús Montoya Ramírez, Rommel Ramírez López, Hugo Fernando Narvaez Gonzalez, Jhon Mauricio Coronel Ruilova, Carlos Felipe Acuña Cota, Juan Sánchez Lora, José Francisco Rodríguez Salinas, Dania Ramírez González, Edaly Cadenas Toledo, Jorge Iván Morales Montesinos

**Affiliations:** 1 Bariatric Surgery, Centro Médico Nacional 20 de Noviembre, Ciudad de México, MEX; 2 General Surgery, Centro Médico Nacional 20 de Noviembre, Ciudad de México, MEX; 3 Gastrointestinal Endoscopy, Centro Médico Nacional 20 de Noviembre, Ciudad de México, MEX; 4 Surgery, Hospital de Especialidades Dr. Belisario Domínguez, Ciudad de México, MEX; 5 Surgery, Hospital General Monte Sinaí, Guayaquil, ECU

**Keywords:** bariatric surgery, comparative study, gastric bypass surgery, multidisciplinary follow-up, roux en y gastric bypass, sleeve gastrectomy, weight-loss outcomes

## Abstract

Introduction

Adherence to multidisciplinary follow-up is considered an important determinant of long-term weight loss after metabolic and bariatric surgery (MBS). In addition, structured preoperative assessment and appropriate selection of surgical technique may influence postoperative outcomes and partly explain differences reported between procedures such as laparoscopic sleeve gastrectomy (LSG) and laparoscopic Roux-en-Y gastric bypass (LRYGB).

Objective

To compare 24-month weight outcomes in patients undergoing LSG and LRYGB within a standardized preoperative and multidisciplinary follow-up framework.

Methods

A retrospective comparative cohort study was conducted, including patients who underwent MBS between January 2021 and December 2023. Outcomes included percentage of total weight loss (%TWL), percentage of excess weight loss (%EWL), insufficient weight loss (%TWL <20%), and weight regain at six, 12, 18, and 24 months.

Results

A total of 71 patients were included (LSG: n=45 (63.4%); LRYGB: n=26 (36.6%)). Baseline characteristics were comparable between groups. At 24 months, no significant differences were observed between LSG and LRYGB in %TWL (29.9% vs. 30.8%) or %EWL (61.6% vs. 63.4%) (p>0.05). Insufficient weight loss, defined as %TWL <20%, decreased from 21.1% at six months to 9.9% at 12 months. Weight regain at 24 months was observed in 25.4% of patients, without significant differences between procedures. No baseline variable included in the model showed a statistically significant association with weight-loss success.

Conclusions

In a clinical setting characterized by standardized preoperative selection and strict multidisciplinary follow-up, LSG and LRYGB showed no statistically significant differences in the 24-month weight-loss outcomes. These findings suggest that patient selection, multidisciplinary follow-up, and institutional factors may contribute to the observed results, although their relative impact requires further investigation.

## Introduction

Severe obesity is a growing global health concern due to its increasing prevalence and its strong association with metabolic and cardiovascular comorbidities, including type 2 diabetes mellitus, hypertension, obstructive sleep apnea, and cardiovascular disease [1]. In this context, metabolic and bariatric surgery (MBS) has demonstrated greater effectiveness than non-surgical interventions in achieving sustained weight reduction and improving obesity-related conditions [2]. Among the most frequently performed procedures worldwide are laparoscopic sleeve gastrectomy (LSG) and laparoscopic Roux-en-Y gastric bypass (LRYGB). While LSG is primarily considered a restrictive procedure, it also exerts hormonal effects, whereas LRYGB combines restrictive and malabsorptive mechanisms, contributing to its metabolic impact [3,4].

Previous randomized trials and meta-analyses have suggested that LRYGB is associated with greater mid- and long-term weight loss than LSG [5-12]. However, outcomes vary considerably across institutions, likely reflecting differences in patient selection, perioperative management, surgical expertise, and adherence to postoperative follow-up protocols. In particular, multidisciplinary follow-up has been identified as an important factor influencing long-term outcomes and the prevention of metabolic and nutritional complications [13-15].

In Mexico, comparative data between these procedures remain heterogeneous and are often influenced by institutional characteristics and the availability of structured multidisciplinary care [16]. The National Medical Center “20 de Noviembre” is a high-specialty referral center with an established multidisciplinary bariatric surgery program, characterized by standardized preoperative protocols, individualized surgical selection, and structured postoperative follow-up.

The primary objective of this study was to compare 24-month weight-loss outcomes, measured as percentage of total weight loss (%TWL) and percentage of excess weight loss (%EWL), between patients undergoing LSG and LRYGB in a clinical setting with standardized preoperative assessment and structured multidisciplinary follow-up.

Secondary objectives included evaluating the rates of insufficient weight loss, weight regain, and identifying potential predictors of weight-loss success during follow-up.

We hypothesized that within a highly standardized, multidisciplinary bariatric program with individualized procedure selection and rigorous postoperative follow-up, 24-month weight-loss outcomes would not differ significantly between LSG and LRYGB.

## Materials and methods

Study design

A retrospective, analytical, and comparative cohort study was conducted to evaluate weight-loss outcomes after LSG and LRYGB among patients with obesity. Data were obtained from institutional electronic and physical medical records, which included comprehensive medical, surgical, and multidisciplinary information.

Ethics approval

This study was reviewed and approved by the Research, Ethics, and Biosafety Committees of the National Medical Center “20 de Noviembre” Instituto de Seguridad y Servicios Sociales de los Trabajadores del Estado (ISSSTE), Mexico City, Mexico (approval number CMN.RPL.139.2025). The study was conducted in accordance with the Declaration of Helsinki, the Regulations of the General Health Law on Health Research of Mexico, and Good Clinical Practice guidelines.

Study population and sample

The study population consisted of consecutive patients treated at the Bariatric Surgery Clinic of the National Medical Center “20 de Noviembre” who underwent LSG or LRYGB between January 2021 and December 2023. Adults aged ≥18 years with documented follow-up at six, 12, 18, and 24 months were included. Exclusion criteria comprised previous bariatric or gastrointestinal surgery, poorly controlled metabolic diseases, incomplete follow-up, or major postoperative complications that could significantly affect weight evolution. After applying the selection criteria, a final sample of 71 patients was obtained: 45 in the LSG group and 26 in the LRYGB group.

Data collection

Collected variables included age, sex, surgical technique, baseline and follow-up body weight, baseline body mass index (BMI), %TWL, and %EWL at each follow-up time point. Additionally, the following outcomes were evaluated: insufficient weight loss (%TWL <20% at six and 12 months); weight regain (≥10% increase from nadir weight at 24 months); and weight loss success (defined as %TWL ≥ 20% and %EWL ≥ 50%).

Procedures

All patients underwent a standardized multidisciplinary preoperative evaluation. The surgical technique was selected according to predefined institutional criteria.

LRYGB was preferentially indicated in patients with clinically significant gastroesophageal reflux disease, Los Angeles grade II-III esophagitis, hiatal hernia, or severe type 2 diabetes mellitus when a greater metabolic benefit was prioritized. In patients with type 2 diabetes mellitus, the Individualized Metabolic Surgery (IMS) score was incorporated into the decision-making process. Patients with an intermediate probability of metabolic remission according to the IMS score were preferentially considered for LRYGB because of its superior metabolic efficacy. Conversely, in patients with advanced metabolic disease and a low predicted likelihood of remission regardless of procedure type, LSG was often favored to minimize the risks of long-term malabsorption, micronutrient deficiencies, and protein-calorie malnutrition while still providing substantial weight-loss benefit.

LSG was preferentially selected in patients with extreme obesity, particularly those with a BMI greater than 50 kg/m², abdominal wall hernias, reduced anticipated adherence to long-term follow-up, middle-to-low socioeconomic status, chronic kidney disease, chronic liver disease, metabolic dysfunction-associated steatotic liver disease, previous transplantation, history of malignancy, autoimmune or rheumatologic disorders, chronic use of immunomodulatory medications, unavoidable long-term use of nonsteroidal anti-inflammatory drugs (NSAIDs), or chronic tobacco use. In these settings, LSG was favored because of its lower risk of malabsorption, protein-calorie malnutrition, micronutrient deficiencies, marginal ulceration, and interference with medication absorption.

All procedures were performed by a single experienced bariatric surgeon within a standardized institutional bariatric surgery program. LSG was performed over a 40-Fr bougie beginning approximately 4-5 cm proximal to the pylorus. Staple-line reinforcement was not routinely performed. Intraoperative leak testing was carried out using a hydropneumatic technique, and a 19-Fr Blake drain was routinely placed at the end of the procedure.

LRYGB was performed over a 36-Fr bougie with construction of a gastric pouch measuring approximately 4-5 cm in length. The procedure included a 100-cm alimentary limb and a 150-cm biliopancreatic limb. Reconstruction was performed using a simplified antecolic, antegastric technique with a parallel gastrojejunal anastomosis and inferosuperior closure using 3-0 polypropylene suture. Intraoperative leak testing was routinely performed using a hydropneumatic technique, and a 19-Fr Blake drain was routinely placed at the end of the procedure.

Postoperative follow-up included MBS visits for drain removal on postoperative days four to five, followed by evaluations at one, three, and six months, and every six months thereafter until completion of 24 months. Nutritional and psychological follow-up was scheduled at one month, three months, and every six months until 24 months, with additional visits performed when clinically indicated. Body weight was measured during in-person visits using a bioimpedance scale (InBody 770, InBody Co., Seoul, South Korea).

Only patients with complete documented follow-up at six, 12, 18, and 24 months were included in the present analysis. Patients who discontinued follow-up before six months postoperatively were excluded from the study cohort and recorded in a separate institutional database designed for independent analyses of follow-up adherence.

Statistical analysis

Descriptive statistics were used to characterize the cohort. Continuous variables were expressed as mean ± standard deviation (SD) or median (interquartile range), as appropriate. Normality was assessed using the Shapiro-Wilk test.

Between-group comparisons for continuous variables were performed using Student’s t-test for independent samples. Categorical variables were compared using the chi-square test or Fisher’s exact test, as appropriate based on expected cell frequencies. For variables with more than two categories, such as obesity class, a global comparison between groups was performed using the Fisher-Freeman-Halton exact test with Monte Carlo simulation. Longitudinal weight evolution was analyzed using repeated-measures analysis of variance (ANOVA).

A multivariable logistic regression model was constructed to identify independent predictors of weight-loss success, defined as achievement of both %TWL ≥20% and %EWL ≥50% at 24 months. Variables entered into the model included surgical technique, age, sex, and baseline BMI. Odds ratios (ORs) were calculated with corresponding p-values. All p-values were two-tailed and are reported in the corresponding tables. Statistical significance was defined as p<0.05.

All statistical analyses were performed using IBM SPSS Statistics for Windows, Version 26 (Released 2019; IBM Corp., Armonk, New York, United States). Data organization and management were conducted using Microsoft Excel for Mac version 16.99 (Microsoft Corp., Redmond, WA, USA).

## Results

Baseline characteristics

The mean age of the cohort was 45.3 ± 9.0 years, with a predominance of female patients (n=55, 77.5%). Mean baseline body weight was 122.7 ± 30.9 kg, and mean baseline BMI was 45.4 ± 7.5 kg/m². No statistically significant differences were observed between the LSG and LRYGB groups in baseline variables (p>0.05) (Table 1).

**Table 1:** Baseline characteristics of the study population according to surgical technique Data are presented as mean ± standard deviation (SD) or n (%). Continuous variables were compared using Student’s t-test. Categorical variables were compared using the chi-square test or Fisher’s exact test, as appropriate. The obesity class was analyzed using the Fisher–Freeman–Halton exact test with Monte Carlo simulation, and a single global p-value is reported. LSG, laparoscopic sleeve gastrectomy; LRYGB, laparoscopic Roux-en-Y gastric bypass; BMI, body mass index.

Variable	LSG (n = 45)	LRYGB (n = 26)	p-value
Age (years)	44.6 ± 9.3	46.5 ± 8.5	0.402
Female, n (%)	34 (75.6)	21 (80.8)	0.830
Weight (kg)	125.4 ± 33.3	118.1 ± 26.1	0.315
BMI (kg/m²)	45.6 ± 7.7	45.0 ± 7.1	0.760
Obesity class, n (%)			0.871
Class I	4 (8.9)	2 (7.7)	—
Class II	7 (15.6)	3 (11.5)	—
Class III	25 (55.6)	15 (57.7)	—
Super obesity (BMI ≥ 50 kg/m²)	8 (17.8)	6 (23.1)	—
Super-super obesity (BMI ≥ 60 kg/m²)	1 (2.2)	0 (0.0)	—

Overall weight loss trajectory

Weight loss increased progressively up to 18 months and stabilized at 24 months. In the overall cohort, the mean %TWL reached 30.2 ± 9.9%, and the mean %EWL reached 62.3 ± 21.0% at 24 months.

Comparison between surgical techniques

**Figure 1:**
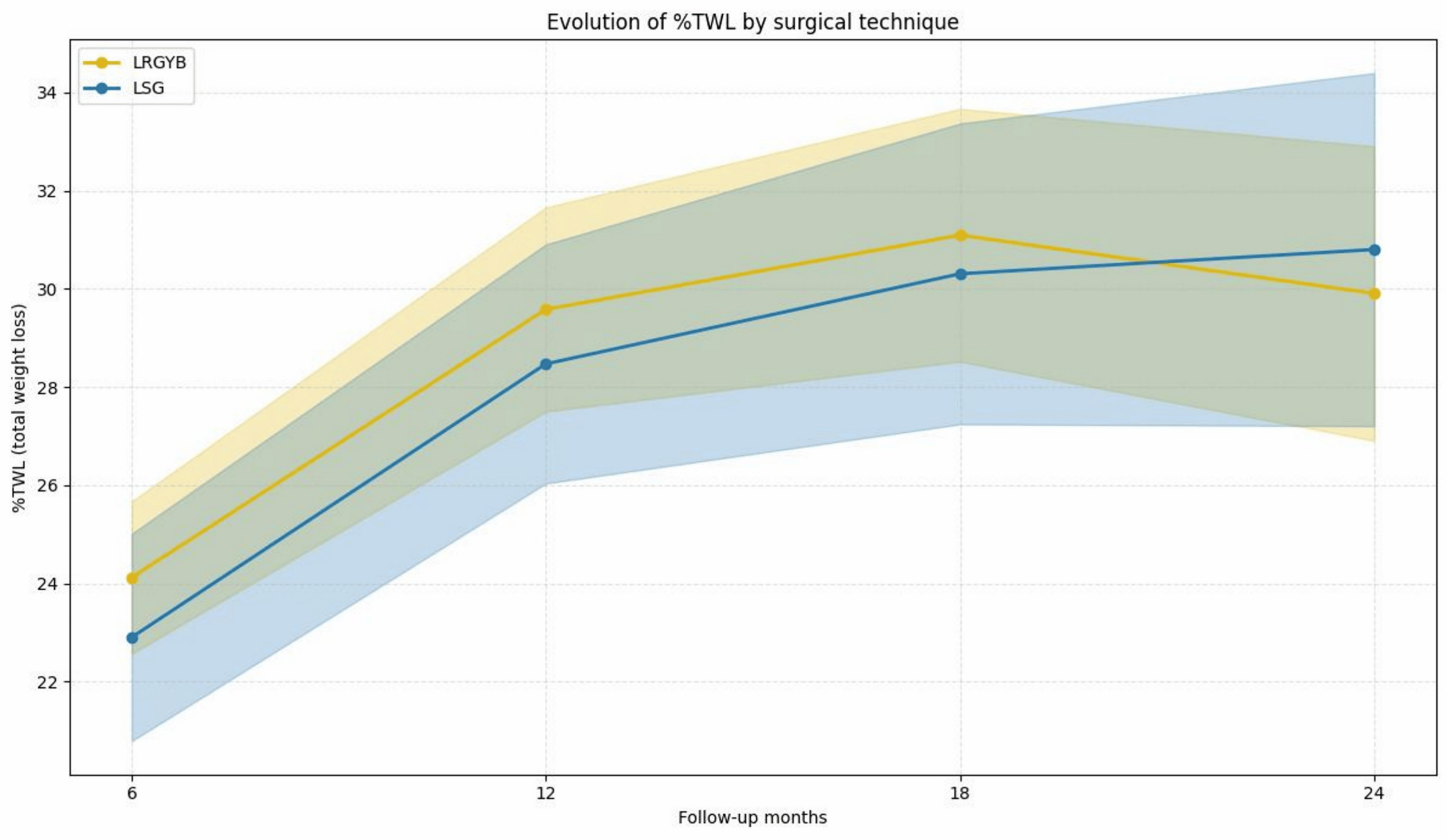
Evolution of percentage of total weight loss (%TWL) in patients undergoing LSG and LRYGB at six, 12, 18, and 24 months of follow-up Both surgical techniques showed a progressive increase in %TWL through 18 months, followed by stabilization at 24 months. Shaded areas represent the standard deviation (SD) at each follow-up time point. No statistically significant differences in %TWL trajectories were detected between LSG and LRYGB during the study period. %TWL, percentage of total weight loss; LSG, laparoscopic sleeve gastrectomy; LRYGB, laparoscopic Roux-en-Y gastric bypass; SD, standard deviation.

No statistically significant differences were observed between LSG and LRYGB in %TWL or %EWL at any follow-up time point. The longitudinal evolution of %TWL across follow-up time points is shown in Figure 1.

**Figure 2:**
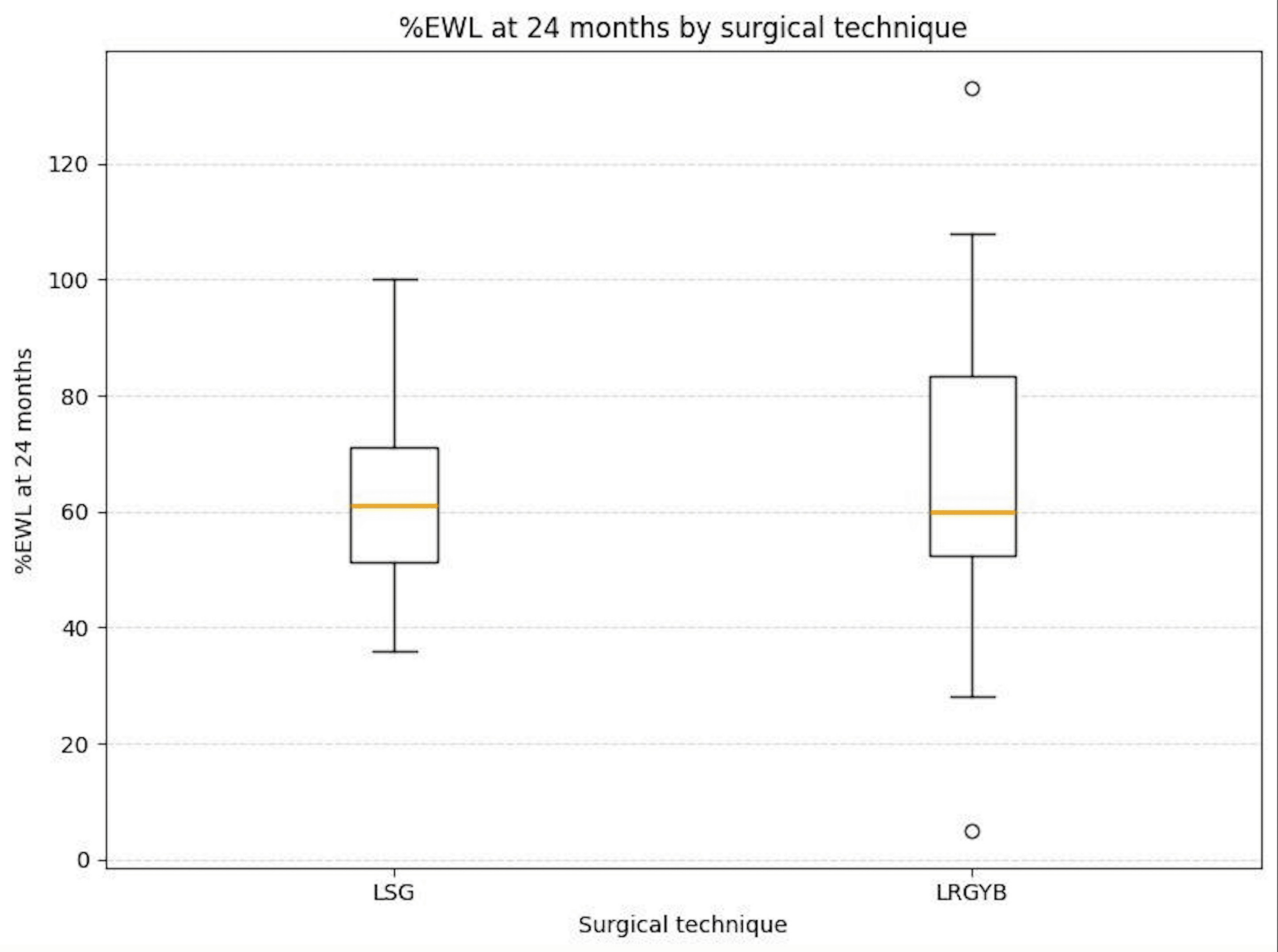
Percentage of excess weight loss (%EWL) at 24 months according to the surgical technique Distribution of percentage of excess weight loss (%EWL) at 24 months among patients undergoing laparoscopic sleeve gastrectomy (LSG) and laparoscopic Roux-en-Y gastric bypass (LRYGB). Median values are represented by the central horizontal line, boxes indicate the interquartile range, and whiskers represent the range of non-outlier observations. No statistically significant difference was observed between groups (p=0.700).

%TWL at 24 months was 29.9% in the LSG group and 30.8% in the LRYGB group (p=0.709). %EWL at 24 months was 61.6% (LSG) vs. 63.4% (LRYGB), p=0.700 (Figure 2).

Repeated-measures ANOVA demonstrated significant changes in weight loss over time in both groups (p<0.001), with no significant interaction between surgical technique and time.

Insufficient weight loss and weight regain

The rate of insufficient weight loss was 21.1% (n=15) at six months and decreased to 9.9% (n=7) at 12 months, with no significant differences between surgical techniques. Weight regain occurred in 25.4% (n=18) of patients at 24 months, without significant differences between groups.

Predictors of weight loss success

Surgical technique, age, sex, and baseline BMI were not significant predictors of weight loss success (p>0.05) (Table 2).

**Table 2:** Logistic regression model for predictors of weight loss success (%TWL ≥20% and %EWL ≥50%) Odds ratios are presented from a multivariable logistic regression model evaluating factors associated with weight-loss success at 24 months. None of the variables included in the model demonstrated a statistically significant association with the outcome (p>0.05). OR, odds ratio; BMI, body mass index; %TWL, percentage of total weight loss; %EWL, percentage of excess weight loss; LSG, laparoscopic sleeve gastrectomy; LRYGB, laparoscopic Roux-en-Y gastric bypass.

Variable	OR	p-value
Surgical technique (LRYGB vs. LSG)	0.85	0.78
Sex	2.34	0.22
Age	0.98	0.62
Baseline BMI	0.99	0.82

## Discussion

In the present study, LSG and LRYGB demonstrated similar weight-loss outcomes at 24 months when performed within a highly standardized institutional framework characterized by rigorous preoperative assessment, individualized procedure selection, refined operative technique, and structured multidisciplinary follow-up. These findings contrast with several randomized trials and meta-analyses reporting greater mid- and long-term weight loss with LRYGB [5-9]. It should be emphasized that the absence of statistically significant differences should not be interpreted as evidence of formal equivalence between procedures, as the study was not designed or powered to test equivalence or non-inferiority. Rather, the findings indicate that no statistically significant differences were detected in this institutional bariatric program.

The discrepancy between our results and those of larger international series may be explained by contextual and system-related factors rather than intrinsic procedural differences. In many comparative studies, variability in patient selection criteria, perioperative protocols, surgical expertise, and long-term follow-up practices may contribute to divergent outcomes. In our cohort, all included patients completed the predefined follow-up evaluations at six, 12, 18, and 24 months, allowing consistent longitudinal assessment. Although all included patients completed the scheduled follow-up visits, the quality, intensity, and behavioral adherence to nutritional and psychological recommendations were not objectively quantified. Structured nutritional and psychological surveillance may have contributed to the favorable outcomes observed in this cohort. However, because adherence was not formally measured and no comparison group without structured follow-up was available, the present study cannot determine the independent effect of multidisciplinary follow-up on weight-loss outcomes. Therefore, these observations should be interpreted as hypothesis-generating rather than evidence of a causal relationship [10-13].

Another relevant consideration is the individualized selection of surgical technique. Procedure allocation was based on predefined clinical, metabolic, anatomical, and social criteria, which may have optimized patient-procedure matching and reduced outcome variability. Rather than comparing techniques in isolation, these findings suggest that outcomes may be influenced by the appropriateness of procedural selection within a given clinical context. Nevertheless, because of the observational design and the possibility of residual selection bias, the relative contribution of procedure selection to the observed outcomes cannot be fully determined.

Both procedures demonstrated progressive weight loss up to 18 months, followed by stabilization at 24 months, a pattern consistent with previously described bariatric weight trajectories. The absence of a significant interaction between surgical technique and time suggests that no statistically significant differences in weight-loss trajectories were detected during the follow-up period. However, the relatively small sample size, particularly in the LRYGB group, may have limited the statistical power to identify modest differences between procedures.

The absence of significant predictors of weight-loss success should also be interpreted with caution. Surgical technique, age, sex, and baseline BMI were not independently associated with achievement of combined %TWL ≥20% and %EWL ≥50%. However, the limited number of variables available for analysis and the retrospective nature of the dataset may have prevented identification of other clinically relevant predictors, including behavioral, metabolic, and adherence-related factors.

Our findings should also be interpreted in the context of previously published Mexican MBS series. Lomelí-Reyes et al. reported greater long-term weight loss after LRYGB compared with LSG in a Mexican population, whereas Pereyra-Talamantes et al. demonstrated favorable metabolic outcomes following both procedures. The absence of significant differences in the present study may reflect differences in patient selection, institutional protocols, surgical standardization, and follow-up practices. These observations highlight the potential influence of local practice patterns and organizational factors on postoperative outcomes [14-16].

The magnitude of weight loss observed in this cohort falls within ranges reported in the international literature, with relatively low rates of insufficient weight loss and weight regain at 24 months. These findings demonstrate that favorable outcomes can be achieved with either procedure within a highly organized bariatric program. However, whether the observed similarity in outcomes reflects true attenuation of procedural differences, limited statistical power, selection factors, or specific institutional characteristics remains uncertain and warrants evaluation in larger prospective multicenter studies.

Strengths and limitations

This study was conducted in a high-specialty institutional setting with standardized preoperative protocols, individualized surgical selection, refined operative techniques, and structured multidisciplinary follow-up through 24 months. The longitudinal assessment at multiple time points strengthens internal consistency and allows evaluation of weight trajectories. Baseline comparability between groups supports appropriate inter-procedural comparison, and the use of clearly defined outcome criteria enhances reproducibility.

However, the retrospective design limits causal inference and introduces potential selection bias. The absence of randomization may have further contributed to selection bias despite standardized selection criteria. In addition, because only patients with complete documented follow-up at six, 12, 18, and 24 months were included, the study cohort may not fully represent the entire population undergoing MBS during the study period. No a priori sample size calculation was performed because of the retrospective nature of the study, and all eligible patients meeting the inclusion criteria were included. Consequently, the relatively small sample size, particularly in the LRYGB group, may have limited the statistical power to detect small-to-moderate differences between procedures. Furthermore, because the study was not designed as a non-inferiority or equivalence trial, the absence of statistically significant differences should not be interpreted as evidence of equivalence between LSG and LRYGB. Follow-up was limited to 24 months, and longer-term outcomes were not assessed. Additionally, behavioral and adherence-related variables were not objectively measured, metabolic outcomes were not systematically analyzed, and the number of variables included in the predictive model was limited by the retrospective design and available dataset. Consequently, potentially relevant predictors of long-term weight-loss success may not have been captured.

Prospective studies with larger sample sizes, longer follow-up periods, objective assessment of adherence to follow-up, and incorporation of behavioral and metabolic parameters are warranted to further clarify the relative contributions of surgical technique and multidisciplinary care to long-term outcomes after MBS.

## Conclusions

These findings indicate that, within a single-center bariatric program characterized by standardized preoperative assessment, individualized procedure selection, refined surgical technique, and structured multidisciplinary follow-up, no statistically significant differences in %TWL or %EWL were observed between LSG and LRYGB at 24 months.

Because of the retrospective design, limited sample size, and absence of formal equivalence testing, these findings should not be interpreted as evidence of equivalence between procedures. Whether the observed similarity in outcomes reflects true attenuation of procedural differences, limited statistical power, selection factors, or specific institutional characteristics remains to be determined in larger prospective studies.
